# Secular trend of gestational diabetes mellitus and its interaction effect with advanced maternal age on adverse maternal-perinatal outcomes among primiparous singleton and twin pregnancies in Hubei, China (2011-2019)

**DOI:** 10.3389/fendo.2025.1439592

**Published:** 2025-09-02

**Authors:** Nawsherwan, Hong-tao Shen, Ijaz Ul Haq, Ghulam Nabi, Shafaq Naeem, Jie-lian Xu, Xiaoqiu Ni

**Affiliations:** ^1^ Xiamen Cardiovascular Hospital of Xiamen University, School of Medicine, Fujian Branch of National Clinical Research Center for Cardiovascular Diseases, Xiamen, China; ^2^ Pharmacy Department, The Affiliated Jiangning Hospital of Nanjing Medical University, Nanjing, Jiangsu, China; ^3^ Department of Clinical Nutrition, College of Applied Medical Sciences, King Faisal University, Al-Ahsa, Saudi Arabia; ^4^ School of Food and Biological Engineering, Jiangsu University, Zhenjiang, China; ^5^ Department of Preventive Medicine, School of Public Health, Wuhan University, Wuhan, China; ^6^ Clinical Nutrition Department, The Affiliated Jiangning Hospital of Nanjing Medical University, Nanjing, Jiangsu, China; ^7^ Department of Traditional Chinese Medicine, The First Affiliated Hospital of Wenzhou Medical University, Wenzhou, Zhejiang, China

**Keywords:** singleton, twins, gestational diabetes mellitus, advanced maternal age, adverse outcomes

## Abstract

**Background:**

Gestational diabetes mellitus (GDM) is a metabolic disorder of pregnancy associated with multiple adverse maternal-perinatal outcomes among singleton and twin pregnancies and its incidence is increasing across the globe. We aimed to find the secular trend of GDM and its interaction effect with advanced maternal age (AMA) on adverse maternal-perinatal outcomes among primiparous singleton and twin pregnancies in Hubei, China.

**Methods:**

A retrospective-based cohort study was conducted at the Wuhan University Renmin Hospital, Hubei Province, China, between 2011 and 2019. A chi-square test was used to explore a significant difference in the adverse maternal-perinatal outcomes between younger (18–34 years) and older/AMA (≥35 years) women with singleton and twin gestations. A multiple binary logistic regression model was used to estimate the adverse effect of GDM on maternal-perinatal outcomes among younger and older women with singleton and twin gestations, taking non-GDM as a reference group. We used a joinpoint regression analysis to find the secular trend of GDM prevalence among singleton and twin pregnancies during the study period.

**Results:**

The secular trend of GDM [average annual percentage change (AAPC) 51.3% (95% confidence interval (95%CI): 3.9, 120.5)] significantly increased among singleton pregnant women. Based on age groups, the secular trend of GDM significantly increased in younger women with singleton [AAPC, 53.2% (95%CI: 2.0, 130.0)] and twin gestations [AAPC, 83.7% (95%CI: 36.0, 148.1)] between 2011 and 2019. Among younger women with singleton gestation, GDM showed a higher risk of hypertensive disorders of pregnancy (HDP), C-section, and macrosomia compared with non-GDM. Among younger women with twin gestations, GDM increased the risk of nuchal cord, polyhydramnios, and preterm births. GDM was associated with an increased risk of HDP, nuchal cord, macrosomia, and congenital defects among older women with singleton gestation. The interaction effect between GDM and AMA significantly increased the risk of HDP (adjusted odds ratio (aOR), 2.5; 95% CI: 1.8, 3.6), C-section (aOR, 2.5; 95% CI: 1.9, 3.4), and preterm birth (aOR, 1.5; 95% CI: 1.1, 1.9) among singleton pregnancies.

**Conclusion:**

Among younger women with singleton and twin gestations, the secular trend of GDM significantly increased between 2011 and 2019. Among singleton pregnancies, GDM is associated with an increased risk of several adverse maternal-perinatal outcomes in both younger and older women. The interaction effect between GDM and AMA significantly increased the risk of HDP, C-section, and preterm birth among singleton pregnancies.

## Introduction

Gestational diabetes mellitus (GDM) is a metabolic disorder of pregnancy, characterized by hyperglycemia that is first detected in pregnancy. It can affect up to 25% of women during pregnancy (1) and its incidence rate varies across different regions of the world including South-East Asia (25%), Middle East and North Africa (17.5%), Europe (12.6%), and North America and the Caribbean region (10.4%) (2, 3). In China, the pooled GDM prevalence was 14.8% in 2019 and its prevalence varies across different cities and regions (4). The prevalence of GMD was 24.2% in Beijing (5), 19.9% in Xiamen (6), 22.9% in Guangzhou (7), 15.8% in Chengdu (8), and 5.1% in Xinjiang (4). According to the American Diabetes Association, the temporal trend of GMD incidence significantly increased by 85% in China (2001–2016) (7). Moreover, the temporal trend of GMD increased by 28% in Xiamen (2012-2017) (6), by 18% in Beijing (2013-2018) (5), and substantially increased in Hubei from 0.6% to 11.8% during 2011-2019 (9) and in Hebei from 3.0% to 15.0% between 2014 and 2021 (10).

The rising incidence rate of GMD could be attributed to the higher prevalence of obesity and increasing childbearing age in China (11, 12). The mean childbearing age has increased from 27.1 years to 29.7 in Hubei, China (11). In China, the proportion of women with advanced maternal age (AMA, ≥35 years) ranges from 10.0% to 20.2% and the proportion of women with AMA increased by 75% and 223% between 2011 and 2019 and during 2010–2017 respectively, in Hubei, China (11, 13). It is well known that both GDM and AMA are independent risk factors associated with a higher risk of hypertensive disorders of pregnancy (HDP), C-section, placenta previa, congenital defects, perinatal mortality, preterm births, and low birth weight (LBW) (8, 9, 12, 14–17). Several studies observed the independent effect of GDM and AMA on adverse maternal-perinatal outcomes (8–10, 12, 14–17) however, very few studies have determined the combined effect of GDM and AMA on maternal-perinatal outcomes (18–21).

Moreover, these previous studies have focused on pooled primiparous and multiparous pregnant women (18–21), thus data on the GDM and its interaction effect with AMA on adverse maternal-perinatal outcomes among primiparous women is limited (22, 23). The pregnancy outcomes of primiparous women are significantly different than multiparous women and represent a unique and pivotal period for assessing various medical, psychological, and social outcomes without the potential confounding factors introduced by prior births (24, 25). Therefore, to understand the unique pregnancy outcomes of primiparous women, without the bias that may arise from previous births, we aimed to explore the secular prevalence of GDM and the interaction effect of GDM with AMA on adverse maternal-perinatal outcomes among primiparous singleton and twin pregnancies in Hubei, China.

## Materials and methods

### Study design and population

The present retrospective-based cohort study was conducted in the Department of Obstetrics and Gynecology at Wuhan University Renmin Hospital in Hubei, China according to the strengthening of the reporting of observational studies in epidemiology (STROBE) guidelines (26) between 2011 and 2019. The skilled nurses collected and recorded the data on pregnant women (n=25678) in the obstetrics register and electronic database during their examinations. Based on maternal age, pregnant women were divided into younger (18–24 years) and advanced maternal age (AMA) (≥35 years) groups. Furthermore, according to the results of the 75 g oral glucose tolerance test, both younger and older women were allocated to gestational diabetes mellitus (GDM) and non-GDM groups. The study protocol was approved by the Ethical Review Board of Renmin Hospital (ID: WDRY2019–K034) *in accordance with the Declaration of Helsinki.*

### Inclusion and exclusion criteria and sample size calculation

**Figure 1:**
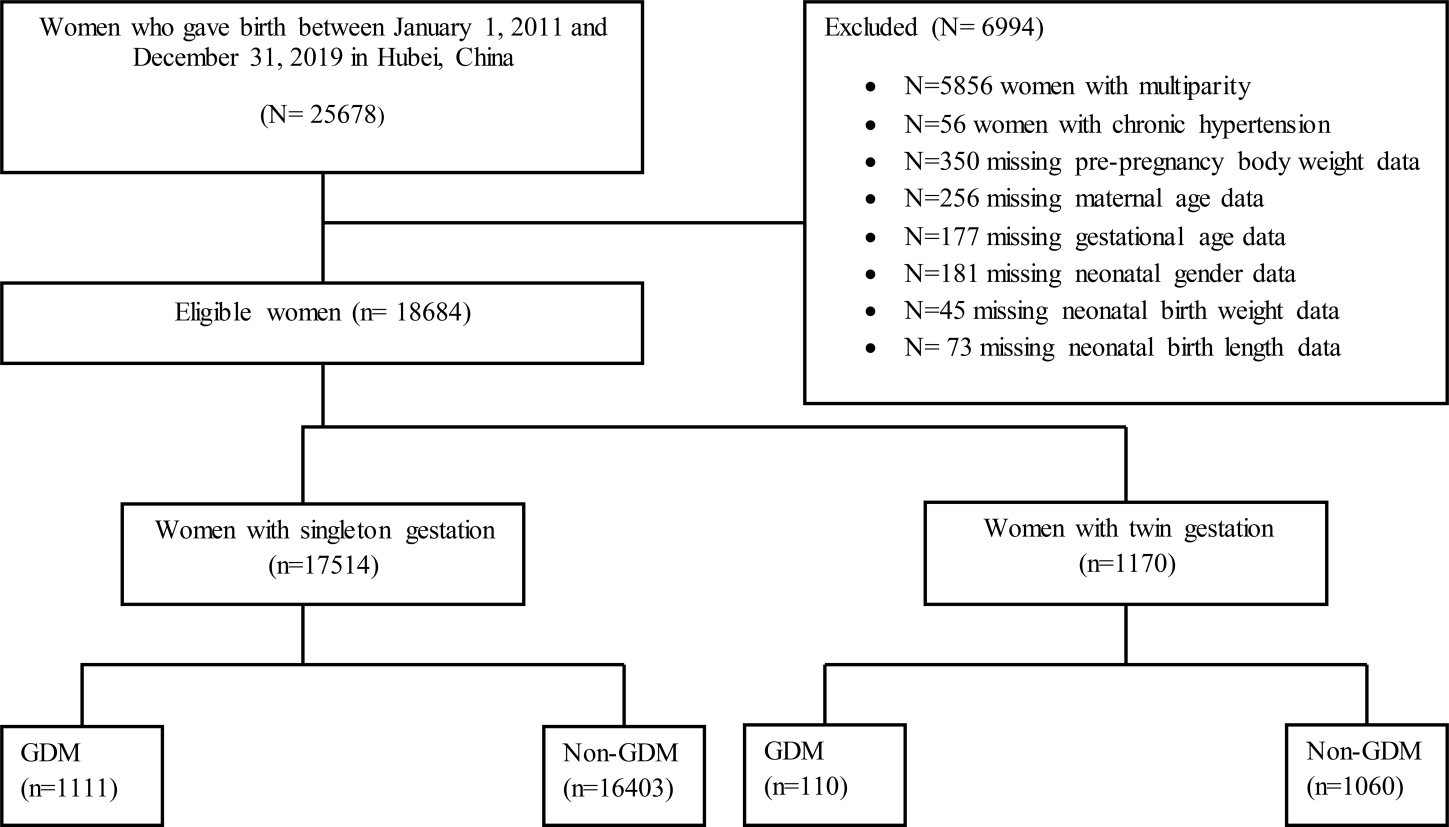
Flow chart of the study population.

A total of 18,684 primiparous pregnant women were considered for data analysis in the current study, including singleton (n= 17514) and twin pregnancies (n= 1170). The data was excluded on multiparty (n= 5856), chronic hypertension (n=56), and missing data on maternal age (n=256), pre-pregnancy body weight (n=350), gestational age (n=177), and neonatal-related data including neonatal gender (n=181), birth weight (n=45), and birth length (n=73) from the statistical analysis as shown in **Figure 1**. In our study, the pattern of missing data was missing at random (MAR) and we applied the listwise or case deletion approach for handling missing data which simply means eliminating the subjects with missing data and analyzing the remaining data (27). The power of the study and sample size was determined by using the G*Power software (latest ver. 3.1.9.7; Heinrich-Heine-Universität Düsseldorf, Düsseldorf, Germany) (28). We used *a priori* power method by selecting Z tests (i.e. test family) and logistic regression (i.e. statistical test) to find the power analysis. We assumed that 30% of non-GDM pregnant women had adverse maternal-perinatal outcomes and 50% of pregnant women with GDM had adverse maternal-perinatal outcomes. By considering the aforementioned assumptions and taking the power (1-β =0.80) and significance level (α =0.05), the minimum sample size should be (N=191).

### Collection of data, variables, and definitions

Data on maternal characteristics including age, pre-pregnancy body weight, education, occupation gestational age, and parity, and maternal-neonatal outcomes such as GDM, hypertensive disorders of pregnancy (HDP), abnormal placentation, previous history of C-section, C-section, premature rupture of membrane (PROM), nuchal cord, oligohydramnios, polyhydramnios, fetal breech presentation, preterm birth, low birth weight (LBW), perinatal mortality, macrosomia, low Apgar score, fetal distress, intrauterine growth restriction (IUGR), congenital defects, neonatal birth weight, birth length, and gender were retrieved from the obstetrics register and electronic database. Maternal age was divided into two groups (i) 18–34 years, (ii) and ≥35 years at the time of delivery. The last known menstrual cycle date was used to determine gestational age, which was then verified by ultrasound examination in the first and second trimesters. The detailed definition of exposure and outcome variables has been described in our previous article (9) and **Supplementary File S1**.

### Definition of confounding factors

Confounding variables that have been linked to both exposure and perinatal birth outcome in the previous literature were selected such as maternal education, occupation, pre-pregnancy body weight (≤ 45 kg and ≥ 91 kg), and neonatal gender (29).

**Statistical analysis**

In the first step, we explored a significant difference in the baseline general characteristics and maternal-perinatal outcomes between younger (18–34 years) and older (≥35 years) primiparous singleton and twin pregnancies by using a Chi-square test. In the second step, multiple binary logistic regression models were used (adjusted for maternal education, occupation, pre-pregnancy body weight, and neonatal gender) to estimate the adverse effect of GDM on maternal-perinatal outcomes among younger and older pregnant women in both singleton and twin pregnancies taking non-GDM as a reference group. In our analysis, GDM was a predictor variable and the outcome variables were HDP, abnormal placentation, C-section, PROM, nuchal cord, oligohydramnios, polyhydramnios, fetal breech presentation, preterm birth, LBW, perinatal mortality, macrosomia, low Apgar score, fetal distress, IUGR, and congenital defects. Furthermore, among singleton and twin pregnancies, we determined the interaction effect of GDM and AMA (i.e. GDM×AMA) on maternal-perinatal outcomes. The adjusted odds ratios (aOR) with 95% confidence intervals were used to estimate the association between predictor variables and outcome variables. *P-value* (two-tailed <0.05) was taken as statistically significant. The data were analyzed by using SPSS (Statistical Package for Social Sciences) for Windows version 22 (IBM Corporation, New York, USA).

In the third step, we used joinpoint regression analysis to find the secular trend of GDM prevalence among primiparous singleton and twin pregnancies between 2011 and 2019. The percentage changes (APC) and average annual percentage changes (AAPC) were estimated for GDM prevalence in the joinpoint regression analysis. The APC shows the trend in the GDM prevalence in each segment and the AAPC indicates trend in the GDM prevalence in the whole study period 2011-2019. The APPC or APC >0 with its 95% confidence interval (CI) shows a positive secular trend, however, the APPC or APC <0 with its 95% CI shows a negative secular trend. Moreover, Monte Carlo methods were used to find each *p-value* and maintain the overall asymptotic significance level through Bonferroni correction. The joinpoint regression analysis was conducted using the joinpoint regression program version 4.8.0.1 (April 2020) from the Surveillance Research Program of the U.S. National Cancer Institute.

## Results

### Baseline maternal-perinatal characteristics among primiparous singleton and twin pregnancies

A total of 18,684 primiparous pregnant women including singleton (n= 17514) and twins (n= 1170) were considered in the current study (**Figure 1**). Singleton and twin pregnancies consisted of 13.2% and 15.1% of women with advanced maternal age (AMA), respectively. Among singleton, women with AMA showed a significantly higher incidence of GDM (11.7% vs. 5.5%), HDP (8.8% vs. 5.8%), abnormal placentation (5.9% vs. 3.3%), C-section (75.8% vs. 56.5%), preterm birth (23.4% vs. 16.5%), LBW (15.5% vs. 12.7%), and perinatal mortality (2.0% vs. 1.2%) compared with younger women. Moreover, women with AMA showed a significantly higher incidence of preterm birth (78.0% vs. 70.4%) compared with younger women among twin pregnancies (**Tables 1**, **2**).

**Table 1:** Baseline maternal characteristics and pregnancy outcomes among primiparous singleton and twin pregnancies by maternal age groups (N= 18684).

Maternal characteristics and pregnancy outcomes	Singleton (n= 17514)			Twins (n= 1170)		
	18–34 Years (n= 15198)	AMA (n= 2316)	P-value	18–34 Years (n= 993)	AMA (n= 177)	P-value
Maternal education			0.8			0.1
Higher	6277 (41.3)	968 (41.8)		352 (35.4)	66 (37.3)	
Middle	6028 (39.7)	913 (39.4)		394 (39.7)	57 (32.2)	
Low	2893 (19.0)	435 (18.8)		247 (24.9)	54 (30.5)	
Maternal occupation			0.2			0.3
Professional services	7233 (47.6)	1110 (47.9)		418 (42.1)	79 (44.6)	
Manual workers	362 (2.4)	67 (2.9)		20 (2.0)	6 (3.4)	
House wives	7603 (50.0)	1139 (49.2)		555 (55.9)	92 (52.0)	
GDM^*^	839 (5.5)	272 (11.7)	<0.001	92 (9.3)	18 (10.2)	0.6
HDP^*^	883 (5.8)	204 (8.8)	<0.001	120 (12.1)	25 (14.1)	0.4
Abnormal Placentation^*^	507 (3.3)	136 (5.9)	<0.001	25 (2.5)	8 (4.5)	0.1
Previous history of C-section^*^	1259 (8.3)	535 (23.1)	<0.001	64 (6.5)	18 (10.2)	0.07
C-section^*^	8586 (56.5)	1756 (75.8)	<0.001	800 (80.6)	150 (84.7)	0.2
PROM^*^	1473 (9.7)	199 (8.6)	0.09	117 (11.8)	18 (10.2)	0.6
Nuchal cord^*^	739 (4.9)	87 (3.8)	0.01	51 (5.1)	7 (4.0)	0.7
Oligohydramnios^*^	619 (4.1)	75 (3.2)	0.06	6 (0.6)	2 (1.1)	0.3
Polyhydramnios^*^	57 (0.4)	5 (0.2)	0.3	4 (0.4)	2 (1.1)	0.2
Fetal breech presentation^*^	387 (2.5)	68 (2.9)	0.2	27 (2.7)	4 (2.3)	0.7

p-Values were calculated using the chi-square test. *Frequency and percentage of variables with only "yes" value presented; AMA, advanced maternal age ≥35 years); HDP, hypertensive disorders of pregnancy; GDM, gestational diabetes mellitus; PROM, premature rupture of membrane.

**Table 2:** Baseline perinatal outcomes among primiparous singleton and twin pregnancies by maternal age groups.

Perinatal outcomes	Singleton (n= 17514)			Twins (n= 1170)		
	18–34 Years (n= 15198)	AMA (n= 2316)	P-value	18–34 Years (n= 993)	AMA (n= 177)	P-value
Preterm birth*	2503 (16.5)	541 (23.4)	<0.001	699 (70.4)	138 (78.0)	0.04
LBW*	1934 (12.7)	358 (15.5)	<0.001	668 (67.3)	100 (56.5)	0.006
Perinatal mortality*	175 (1.2)	47 (2.0)	<0.001	20 (2.0)	4 (2.3)	0.7
LPI*	562 (3.7)	94 (4.1)	0.3	90 (9.1)	15 (8.5)	0.8
Macrosomia*	812 (5.3)	118 (5.1)	0.6	2 (0.2)	0 (0)	0.5
Low Apgar score*	482 (3.2)	91 (3.9)	0.05	162 (16.3)	23 (13.0)	0.2
Fetal distress*	369 (2.4)	50 (2.2)	0.4	8 (0.8)	2 (1.1)	0.6
IUGR*	111 (0.7)	12 (0.5)	0.2	5 (0.5)	0 (0)	0.3
Congenital defects*	199 (1.3)	30 (1.3)	0.9	5 (0.5)	1 (0.6)	0.9
Neonatal gender			0.6			0.2
Male	7957 (52.4)	1224 (52.8)		502 (50.6)	98 (55.4)	
Female	7241 (47.6)	1092 (47.2)		491 (49.4)	79 (44.6)	

p-values were calculated using the chi-square test. *Frequency and percentage of variables with only "yes" value presented; LBW, low birth weight; IUGR, intrauterine growth restriction; LPI, low ponderal index; *congenital defects [microtia, anotia, polydactyly, heart defects, limb reduction defects, cleft lip, cleft palate, hydrocephaly, and neural tube defects (NTDs)].

### Association of GDM with adverse maternal–perinatal outcomes among younger and older women with singleton gestation

After adjusting for confounding factors, GDM was associated with a higher risk of HDP (aOR, 2.1; 95% CI: 1.6, 2.5), C-section (aOR, 1.6; 95% CI: 1.4, 1.9), and macrosomia (aOR, 1.4; 95% CI: 1.1, 1.8) compared with non-GDM among younger women aged (18–34 years). Among women with AMA, GDM was associated with an increased risk of HDP (aOR, 1.9; 95% CI: 1.3, 2.7), nuchal cord (aOR, 1.8; 95% CI: 1.1, 3.1), macrosomia (aOR, 1.6; 95% CI: 1.1, 2.7), and congenital defects (aOR, 2.4; 95% CI: 1.1, 5.7) compared with non-GDM (**Table 3**).

**Table 3:** Risk of maternal-perinatal outcomes among primiparous singleton women with gestational diabetes mellitus by maternal age groups.

Maternal-perinatal outcomes	18–34 Years			AMA			Interaction effect
	Non-GDM (n= 14359)	GDM (n= 839)	aOR (95%CI)	Non-GDM (n=2044)	GDM (n= 272)	aOR (95%CI)	GDM×AMA aOR (95%CI)
HDP	791 (5.5)	92 (11.0)	2.1 (1.6, 2.5)	165 (8.1)	39 (14.3)	1.9 (1.3, 2.7)	2.5 (1.8, 3.6)*
Abnormal Placentation	480 (3.3)	27 (3.2)	0.9 (0.6, 1.4)	123 (6.0)	13 (4.8)	0.8 (0.4, 1.4)	1.3 (0.7, 2.3)**
C-section	8015 (55.8)	571 (68.1)	1.6 (1.4, 1.9)	1542 (75.4)	214 (78.7)	1.1 (0.8, 1.6)	2.5 (1.9, 3.4)*
PROM	1421 (9.9)	52 (6.2)	0.6 (0.4, 0.8)	181 (8.9)	18 (6.6)	0.7 (0.4, 1.2)	0.6 (0.4, 1.1)*
Nuchal cord	719 (5.0)	20 (2.4)	0.4 (0.3, 0.7)	71 (3.5)	16 (5.9)	1.8 (1.1, 3.1)	1.2 (0.7, 2.1)**
Oligohydramnios	594 (4.1)	25 (3.0)	0.7 (0.4, 1.0)	70 (3.4)	5 (1.8)	0.5 (0.2, 1.3)	0.4 (0.2, 1.1)**
Polyhydramnios	55 (0.4)	2 (0.2)	0.6 (0.1, 2.4)	4 (0.2)	1 (0.4)	1.9 (0.2, 7.3)	1.1 (0.1, 7.7)**
Fetal breech presentation	367 (2.6)	20 (2.4)	0.9 (0.5, 1.4)	58 (2.8)	10 (3.7)	1.3 (0.6, 2.6)	1.4 (0.7, 2.7)**
Preterm birth	2347 (16.3)	156 (18.6)	1.1 (0.9, 1.4)	478 (23.4)	63 (23.2)	1.1 (0.7, 1.3)	1.5 (1.1, 1.9)*
LBW	1834 (12.8)	100 (11.9)	0.9 (0.7, 1.1)	320 (15.7)	38 (14.0)	0.8 (0.6, 1.2)	1.1 (0.7, 1.5)**
Perinatal mortality	167 (1.2)	8 (1.0)	0.8 (0.4, 1.7)	41 (2.0)	6 (2.2)	1.1 (0.4, 2.6)	1.8 (0.8, 4.1)**
LPI	530 (3.7)	32 (3.8)	1.1 (0.7, 1.5)	84 (4.1)	10 (3.7)	0.9 (0.4, 1.7)	1.1 (0.5, 1.9)**
Macrosomia	749 (5.2)	63 (7.5)	1.4 (1.1, 1.8)	97 (4.7)	21 (7.7)	1.6 (1.1, 2.7)	1.5 (0.9, 2.3)**
Low Apgar score	467 (3.3)	15 (1.8)	0.5 (0.3, 0.9)	82 (4.0)	9 (3.3)	0.8 (0.4, 1.6)	1.1 (0.5, 2.0)**
Fetal distress	353 (2.5)	16 (1.9)	0.7 (0.4, 1.2)	46 (2.3)	4 (1.5)	0.6 (0.2, 1.8)	0.6 (0.2, 1.6)**
IUGR	105 (0.7)	6 (0.7)	0.9 (0.4, 2.1)	11 (0.5)	1 (0.4)	0.6 (0.1, 5.1)	0.5 (0.1, 3.6)**
Congenital defects	193 (1.3)	6 (0.7)	0.5 (0.2, 1.2)	23 (1.1)	7 (2.6)	2.4 (1.1, 5.7)	2.1 (0.9, 4.4)**

GDM, gestational diabetes mellitus; PROM, premature rupture of membrane; LBW, low birth weight; IUGR, intrauterine growth restriction; LPI, low ponderal index; aOR, adjusted odds ratios; CI, confidence interval. Adjusted for parity, maternal age, education, occupation, pre-pregnancy body weight, and neonatal gender; non-GDM was taken as a reference group. * Statistical power = 80%, **statistical power < 80%.

### Association of GDM with adverse maternal–perinatal outcomes among younger and older women with twin gestations

Among younger women, individuals with GDM had a higher risk of nuchal cord (aOR, 2.3; 95% CI: 1.1, 5.1), polyhydramnios (aOR, 11.6; 95% CI: 1.5, 18.1), and preterm births (aOR, 1.9; 95% CI: 1.2, 3.3) compared with non-GDM. However, GDM was not significantly associated with an increased risk of any maternal-perinatal outcomes among women with AMA (**Table 4**).

**Table 4:** Risk of maternal-perinatal outcomes among primiparous twin women with gestational diabetes mellitus by maternal age groups.

Maternal-perinatal outcomes	18–34 Years			AMA			Interaction effect
	Non-GDM (n= 901)	GDM (n= 92)	aOR (95%CI)	Non-GDM (n= 159)	GDM (n=18)	aOR (95%CI)	GDM×AMA aOR (95%CI)
HDP	106 (11.8)	14 (15.2)	1.3 (0.7, 2.4)	23 (14.6)	2 (11.1)	0.7 (0.2, 3.9)	0.9 (0.2, 4.1)**
Abnormal Placentation	23 (2.6)	2 (2.2)	0.8 (0.2, 3.5)	7 (4.4)	1 (5.6)	2.2 (0.2, 5.6)	2.2 (0.2, 8.9)**
C-section	728 (80.8)	72 (78.3)	0.8 (0.4, 1.3)	131 (82.9)	17 (94.4)	3.7 (0.4, 9.1)	3.9 (0.5, 9.7)**
PROM	110 (12.2)	7 (7.6)	0.6 (0.2, 1.3)	17 (10.8)	1 (5.6)	0.2 (0.1, 2.8)	0.4 (0.1, 3.2)**
Nuchal cord	42 (4.7)	9 (9.8)	2.3 (1.1, 5.1)	7 (4.4)	0 (0)	0 (0)	0 (0)**
Oligohydramnios	5 (0.6)	1 (1.1)	1.9 (0.2, 7.1)	2 (1.3)	0 (0)	0 (0)	0 (0)**
Polyhydramnios	2 (0.2)	2 (2.2)	11.6 (1.5, 18.1)	2 (1.3)	0 (0)	0 (0)	0 (0)**
Fetal breech presentation	27 (3.0)	0 (0)	0 (0)	4 (2.5)	0 (0)	0 (0)	0 (0)**
Preterm birth	624 (69.3)	75 (81.5)	1.9 (1.2, 3.3)	121 (76.6)	16 (88.9)	3.7 (0.7, 9.9)	3.4 (0.7, 9.7)**
LBW	610 (67.7)	58 (63.0)	0.8 (0.5, 1.2)	91 (57.6)	9 (50.0)	0.7 (0.2, 2.1)	0.4 (0.1, 1.2)**
Perinatal mortality	19 (2.1)	1 (1.1)	0.4 (0.1, 3.6)	3 (1.9)	1 (5.6)	1.6 (0.2, 9.5)	2.1 (0.2, 7.1)**
LPI	84 (9.3)	6 (6.5)	0.6 (0.2, 1.5)	14 (8.9)	1 (5.6)	0.8 (0.1, 8.1)	0.6 (0.1, 4.7)**
Macrosomia	2 (0.2)	0 (0)	0 (0)	0 (0)	0 (0)	0 (0)	0 (0)**
Low Apgar score	148 (16.4)	14 (15.2)	0.8 (0.4, 1.6)	20 (12.7)	3 (16.7)	1.5 (0.3, 6.6)	0.9 (0.2, 3.4)**
Fetal distress	7 (0.8)	1 (1.1)	2.1 (0.2, 8.1)	2 (1.3)	0 (0)	0 (0)	0 (0)**
IUGR	5 (0.6)	0 (0)	0 (0)	0 (0)	0 (0)	0 (0)	0 (0)**
Congenital defects	5 (0.6)	0 (0)	0 (0)	1 (0.6)	0 (0)	0 (0)	0 (0)**

GDM, gestational diabetes mellitus; PROM, premature rupture of membrane; LBW, low birth weight; IUGR, intrauterine growth restriction; LPI, low ponderal index; aOR, adjusted odds ratios; CI, confidence interval. Adjusted for parity, maternal age, education, occupation, pre-pregnancy body weight, and neonatal gender; non-GDM was taken as a reference group. **statistical power < 80%.

### Interaction effect between GDM and AMA on adverse maternal-perinatal outcomes among women with singleton and twin gestations

Among singleton, the interaction effect between GDM and AMA (i.e., GDM×AMA) significantly increased the risk of HDP (aOR, 2.5; 95% CI: 1.8, 3.6), C-section (aOR, 2.5; 95% CI: 1.9, 3.4), and preterm birth (aOR, 1.5; 95% CI: 1.1, 1.9) compared with non-GDM. On the other hand, the interaction effect between GDM and AMA is not significantly associated with an increased risk of maternal-perinatal outcomes among twins (**Tables 3**, **4**).

### Temporal trend of GDM among primiparous singleton and twin pregnancies (2011-2019)

**Figure 2:**
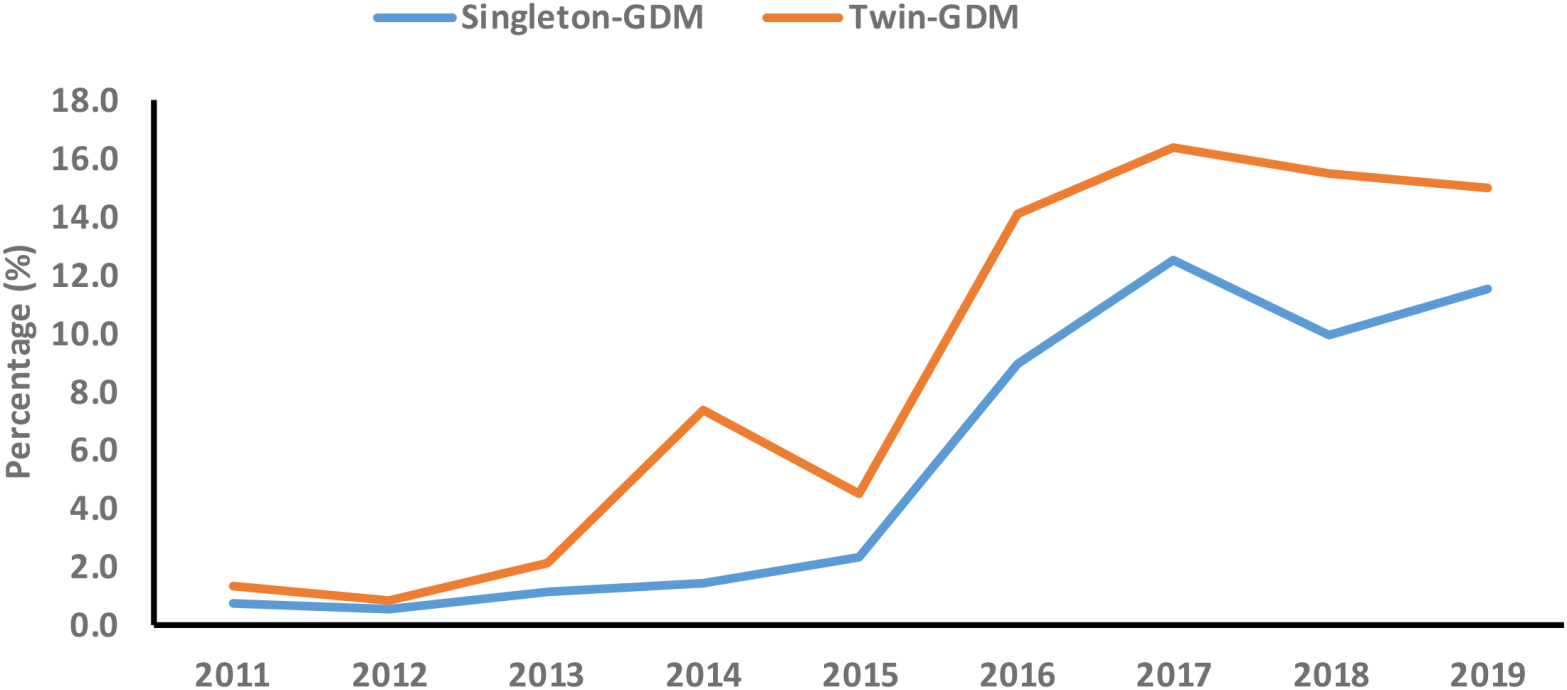
Secular trend of gestational diabetes mellitus (GDM) among primiparous singleton and twin pregnancies (2011-2019).

The joinpoint regression analysis revealed that the APC of GDM significantly increased among primiparous singleton (APC, 70.8%; 95%CI: 23.8, 135.6) and twin pregnancies (APC, 65.3%; 95%CI: 15.0, 137.7) between 2011 and 2017. In addition, the AAPC of GDM significantly increased among primiparous singleton (AAPC, 51.3%; 95%CI: 3.9, 120.5) from 2011 to 2019 (**Table 5**, **Figure 2**).

**Table 5:** Secular trend of GDM among primiparous singleton and twin pregnancies from 2011 to 2019.

Population and variables	Year	APC (95% CI)
Singleton		
Trend1	2011-2017	70.8 (23.8, 135.6)
Trend2	2017-2019	5.3 (-84.3, 605.9)
AAPC (95%CI)	2011-2019	51.3 (3.9, 120.5)
Twins		
Trend1	2011-2017	65.3 (15.0, 137.7)
Trend2	2017-2019	-9.6 (-89.4, 674.3)
AAPC (95%CI)	2011-2019	42.2 (-7.0, 117.5)

GDM, gestational diabetes mellitus; APC, annual percentage change; AAPC, average annual percent change; CI, confidence interval.

### Temporal trend of GDM among primiparous singleton and twin pregnancies by maternal age groups (2011-2019)

**Figure 3:**
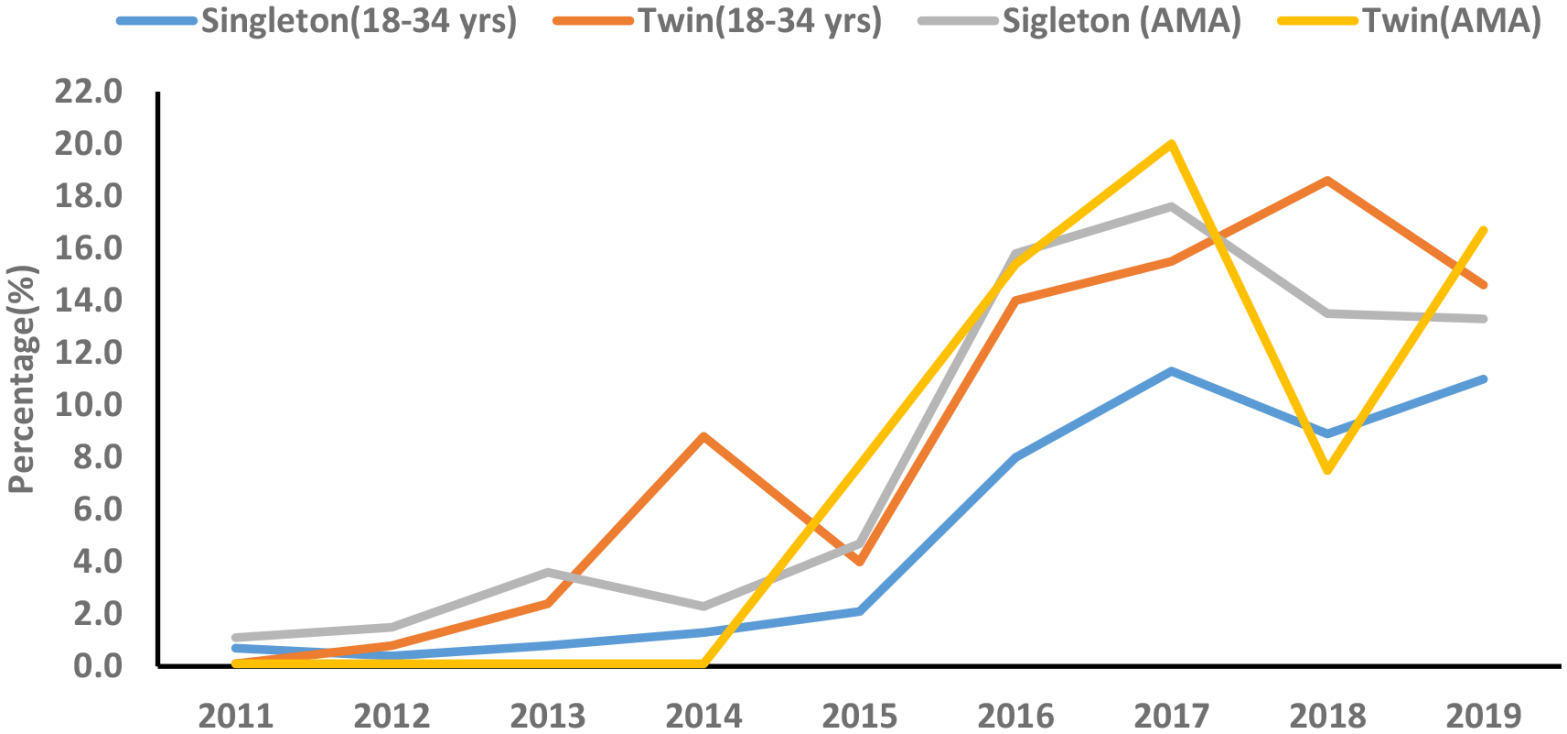
Secular trend of gestational diabetes mellitus (GDM) among primiparous singleton and twin pregnancies by maternal age groups (2011-2019).

The secular trend of GDM significantly increased in both singleton (AAPC, 53.2%; 95%CI: 2.0, 130.0) and twins (AAPC, 83.7%; 95%CI: 36.0, 148.1) younger (18-34 years) women during the study period (2011-2019). Moreover, the secular trend of GDM significantly increased in both singleton (APC, 60.1%; 95%CI: 20.4, 112.9) and twins (APC, 184.8%; 95%CI: 7.0, 658.4) older (≥35years) women between 2011 and 2017 (**Table 6**, **Figure 3**).

**Table 6:** Secular trend of GDM among primiparous singleton and twin pregnancies by maternal age groups from 2011 to 2019.

Population and variables	Year	APC (95% CI)
Singleton (18–34 years)		
Trend1	2011-2017	79.9 (21.5, 143.2)
Trend2	2017-2019	8.4 (-86.1, 745.4)
AAPC (95%CI)	2011-2019	53.2 (2.0, 130.0)
Twins (18–34 years)		
Trend1	2011-2014	280.6 (53.1, 846.2)
Trend2	2014-2019	18.6 (-21.1, 78.3)
AAPC (95%CI)	2011-2019	83.7 (36.0, 148.1)
Singleton (AMA)		
Trend1	2011-2017	60.1 (20.4, 112.9)
Trend2	2017-2019	-12.0 (-83.7, 375.4)
AAPC (95%CI)	2011-2019	37.9 (-1.3, 92.5)
Twins (AMA)		
Trend1	2011-2017	184.8 (7.0, 658.4)
Trend2	2017-2019	-18.4 (-99.8, 2694.5)
AAPC (95%CI)	2011-2019	108.4 (-33.8, 555.9)

GDM, gestational diabetes mellitus; APC, annual percentage change; AAPC, average annual percent change; CI, confidence interval.

## Discussion

In the current retrospective cohort study, we observed that older women had a higher incidence of several adverse maternal-perinatal outcomes compared with younger women in singleton pregnancies. Moreover, older women showed a significantly higher incidence of preterm birth compared with younger women among twin pregnancies. Among singleton pregnancies, GDM is associated with several adverse maternal-perinatal outcomes in both younger and older women. Among twin pregnancies, GDM showed a higher risk of nuchal cord, polyhydramnios, and preterm births in younger women but was not significantly associated with adverse maternal-perinatal outcomes in older women. The additive interaction effect between GDM and AMA significantly increased the risk of HDP, C-section, and preterm birth among singleton pregnancies. The secular trend of GDM significantly increased among singleton pregnancies. Based on age groups, the secular trend of GDM significantly increased in younger women with both singleton and twin gestations during the study period.

### Adverse maternal-perinatal outcomes among singleton and twin pregnancies by maternal age groups

We found that among singleton pregnancies, older women had a significantly higher incidence of GDM, HDP, abnormal placentation, C-section, preterm birth, LBW, and perinatal mortality than younger women. In addition, older women showed a significantly higher incidence of preterm birth compared with younger women among twin pregnancies. Our findings that older women had a higher incidence of adverse maternal-perinatal outcomes than younger women replicate the results of previous studies (13, 22, 23, 30–32). The higher incidence of GDM among older women could be attributed to progressive vascular endothelial damage in older ages (33), impaired glucose tolerance, reduction in insulin sensitivity (34), and pancreatic β-cell dysfunction as maternal age increases (35). Moreover, older women had high oxidative stress and low nitric oxide levels that could be attributed to atherosclerotic changes in the uterine blood vessels and linked with placental vascular pathology (36, 37). Besides these changes, progesterone deficiency in older women may partially explain the higher incidence of maternal-perinatal outcomes in women with AMA (38).

### Association of GDM with adverse maternal–perinatal outcomes among singleton and twin pregnancies by maternal age group

We observed that GDM is associated with an increased risk of several adverse maternal-perinatal outcomes among younger and older women regardless of the number of gestations (i.e. singleton and twins). Among younger women with singleton gestation, GDM was associated with a higher risk of HDP, C-section, and macrosomia compared with non-GDM. Among older women with singleton gestation, GDM increased the risk of HDP, nuchal cord, macrosomia, and congenital defects. Our findings show that GDM increased the risk of HDP and macrosomia among singleton pregnancies regardless of maternal age groups which is in accordance with the previous studies (18, 20). It has been observed that GDM increases the occurrence of gestational hypertension and preeclampsia and insulin resistance plays a significant role in the pathogenies of hypertension during pregnancy (18, 39, 40). We found that among singleton pregnancies, GDM increased the risk of macrosomia by 1.4-fold and 1.6-fold in both younger and older women respectively, compared with non-GDM. The robust association between GMD and neonatal macrosomia was also observed in the previous studies in China (18), the United States (41), Canada (42), and Germany (43). It reflects that fetal exposure to maternal hyperglycemia over a prolonged gestational period resulted in neonatal macrosomia (44).

We showed that among younger women with twin gestations, GDM is associated with a higher risk of nuchal cord, polyhydramnios, and preterm births. However, GDM was not significantly associated with an increased risk of any maternal-perinatal outcomes among older women with twin gestations. It could be partially explained by the small sample size of twin older women with GDM in our study. Several studies observed the adverse maternal-perinatal outcomes in twin pregnancies complicated by GDM (9, 45, 46) or adverse maternal-perinatal outcomes in twin pregnancies by maternal age (32, 47, 48); however, the impact of GDM on adverse maternal-perinatal outcomes among twin pregnancies by maternal age groups has not yet been reported. The present study suggests that a large population-based study should be conducted to investigate the independent effect of GDM on adverse maternal-perinatal outcomes among younger and older twin pregnancies.

### Interaction effect between GDM and AMA on adverse maternal-perinatal outcomes

Our findings show that the interaction effect between GDM and AMA significantly increased the risk of HDP, C-section, and preterm birth among singleton pregnancies. On the other hand, the interaction effect between GDM and AMA is not significantly associated with an increased risk of any adverse maternal-perinatal outcome among twin pregnancies. In accordance with our findings, Li et al. (18) also observed that the interaction effect between GDM and AMA significantly increased the risk of aforementioned adverse maternal-neonatal outcomes among singleton pregnancies. This study (18) investigated the individual effect as well as the interaction effect of GDM and AMA on adverse maternal-neonatal outcomes. However, our study only explored the interaction effect of GDM and AMA on adverse maternal-neonatal outcomes.

Zhang et al. (30) showed that compared with women aged 25–39 years, older women with GDM had a higher risk of several adverse pregnancy outcomes. However, this study failed to determine the interaction effect of AMA and GDM on adverse pregnancy outcomes (30). In another study, Wang et al. (21) investigated the independent effect of GDM and the interaction effect of GDM and maternal age on adverse pregnancy outcomes. The study showed that the interaction effect between GDM and young age (i.e. women aged less than 35 years) significantly increased the risk of C-section among Chinese urban women. However, we haven't explored the interaction effect of GDM and young age on adverse pregnancy outcomes. Overall, these findings suggest that besides the independent effect of GMD and AMA on adverse pregnancy outcomes, future studies should consider the interaction effect between GDM and both young and old age on adverse pregnancy outcomes. Moreover, our findings underscore the significant synergistic effect of GDM and AMA on adverse pregnancy outcomes in singleton women and urge a lifestyle intervention to control blood sugar and minimize the risk of HDP and preterm births among older pregnant women with GDM.

### Secular trend of GDM among primiparous singleton and twin pregnancies (2011–2019)

The secular trend of GDM significantly increased among singleton pregnancies. Based on age groups, the secular trend of GDM significantly increased in younger women with both singleton and twin gestations during the study period (2011–2019). Moreover, the secular trend of GDM significantly increased in older women with singleton and twin gestations between 2011 and 2017. The increasing secular trend of GDM has been observed across different cities and regions in China. Between 2001 and 2016, the secular trend of GDM incidence significantly increased by 85% in China (7). Moreover, the secular trend of GMD increased by 28% in Xiamen (2012–2017) (6), by 18% in Beijing (2013–2018) (5), and substantially increased in Hubei from 0.6% to 11.8% during 2011-2019 (9) and in Hebei from 3.0% to 15.0% between 2014 and 2021 (10). These studies estimated the secular trend of GDM regardless of parity and maternal age groups (5–7, 9, 10). However, our findings show the increasing secular trend of GDM among primiparous younger and older women with singleton and twin gestations. In our study, the increasing trend of GDM among younger women with singleton and twin gestations is alarming and is associated with an increased risk of several adverse maternal-perinatal outcomes. In China, the increasing secular trend of GDM could be attributed to smoking, urbanization, lifestyle changes, pre-pregnancy higher body mass index (BMI), and higher gestational weight gain (6, 49). However, we failed to determine the risk factors associated with the increasing secular trend of GDM in our study due to the lack of certain data including smoking, pre-pregnancy BMI, gestational weight gain, dietary habits, and sedentary lifestyle. Future studies should be encouraged to estimate the secular trend of GDM and its associated risk factors among younger and older women with singleton and twin gestation at the population level in China.

We acknowledge that our study had several limitations. First, our study was a retrospective cohort study conducted in a single-center tertiary hospital, which could be a potential selection bias in our study. Second, our study had missing data and lacked data on maternal smoking, drinking alcohol, dietary habits, sedentary lifestyle, assisted reproductive technology, pre-pregnancy BMI, and gestational weight gain, which may bias our findings. Third, overall we had a small sample size particularly twin older women with GDM, thus the results cannot be generalized to the overall population.

## Conclusion

Among women with singleton gestation, GDM increased the risk of several adverse maternal-perinatal outcomes in both younger and older women. Among women with twin gestations, GDM showed a higher risk of nuchal cord, polyhydramnios, and preterm births in younger women but was not significantly associated with any adverse maternal-perinatal outcomes in older women. The additive interaction effect between GDM and AMA significantly increased the risk of HDP, C-section, and preterm birth among singleton pregnancies. The secular trend of GDM significantly increased in younger singleton and twin pregnancies between 2011 and 2019. Our findings suggest that gynecologists and clinicians should prioritize singleton pregnancies with both GDM and AMA. Additionally, policymakers should implement lifestyle interventions (e.g., physical activity, diet) and ensure timely, free GDM screenings and treatment, particularly for younger women, to address the growing trend of GDM and its impact on maternal and perinatal outcomes.

## Data Availability

The original contributions presented in the study are included in the article/**Supplementary Material**. Further inquiries can be directed to the corresponding authors.
