# Supplementary material for: Secular trend of gestational diabetes mellitus and its interaction effect with advanced maternal age on adverse maternal-perinatal outcomes among primiparous singleton and twin pregnancies in Hubei, China (2011-2019)
Source: Front Endocrinol (Lausanne). 2025 Sep 2;16:1439592. doi: 10.3389/fendo.2025.1439592 (PMC12436103; doi:10.3389/fendo.2025.1439592)
Supplement: Supplementary file 1 [file DataSheet1.docx]

**Definition of exposure and outcome variables**

The exposure variable was gestational diabetes mellitus (GDM). GDM is defined as elevated blood sugar or glucose intolerance during pregnancy which usually disappears after neonatal birth (1). Gestational hypertension (GH) is defined as having blood pressure greater than 140/90 mmHg without proteinuria after the 20^th^ week of gestation (2). Preeclampsia (PE) is defined as elevated blood pressure 140/90 mmHg with proteinuria (albumin > 0.3g in 24 hours) after the 20^th^ week of gestation (3). Severe PE refers to having a blood pressure higher than 160/110 mmHg with proteinuria (albumin > 5g in 24 hours) after the 20^th^ week of gestation (4). The outcome variables are defined as follows. Placenta previa is defined as suboptimal placental implantation near or over the cervical opening (5). Placental abruption refers to the early separation of the placenta before childbirth (6). Neonatal birth outcomes were recorded immediately after neonatal birth including birth weight in grams using an electronic infant scale, and birth length in centimeters using a standard measuring board for the neonate. Preterm birth is defined as a neonate born before 37 completed weeks or fewer than 259 days from the first date of a woman’s last menstrual period (7). Perinatal mortality is defined as the combination of late fetal mortality (stillbirths) and early neonatal mortality (0-6 days of life) (8). Fetal macrosomia is defined as birth weight ≥4000g and low birth weight (LBW) is defined as birth weight < 2500g (9). Intrauterine growth restriction (IUGR) is defined as a condition of fetal growth that is below the 10th percentile for its gestational age and does not reach its genetically predetermined growth potential (10). Apgar score was determined by evaluating the newborn baby on five simple criteria on a scale from zero to two, then summing up the five values obtained. Apgar score was recorded at 1 minute, and at 5 minutes after birth. Apgar score was divided into two categories (i) low Apgar score (<7), and (ii) normal Apgar score (≥ 7) (11). Fetal hypoxia/distress is defined as a pathophysiological condition in which the fetus is suffering from insufficient oxygen supply (12). The ponderal index was determined by weight in gm / (length in cm) ^3^×100. The ponderal index between 2.5 and 3.0 was considered normal, between 2.0 and 2.5 marginal, and a neonate with a ponderal index less than 2.0 was considered a low ponderal index (LPI) (13). A congenital defect is defined as an abnormality in the structure of neonatal body parts that occurs during intrauterine development (14).

**References**

1. Rauh-Hain JA, Rana S, Tamez H, Wang A, Cohen B, Cohen A, et al. Risk for developing gestational diabetes in women with twin pregnancies. *The Journal of Maternal-Fetal Neonatal Medicine* (2009) 22(4):293-9.

2. Program NHBPE. Report of the national high blood pressure education program working group on high blood pressure in pregnancy. *American journal of obstetrics and gynecology* (2000) 183(1):s1-s22.

3. Xiong X, Demianczuk NN, Saunders LD, Wang F-L, Fraser WD. Impact of preeclampsia and gestational hypertension on birth weight by gestational age. *American journal of epidemiology* (2002) 155(3):203-9.

4. Schroeder BM. ACOG practice bulletin on diagnosing and managing preeclampsia and eclampsia. *American family physician* (2002) 66(2):330.

5. Kancherla V, Räisänen S, Gissler M, Kramer MR, Heinonen S. Placenta previa and risk of major congenital malformations among singleton births in Finland. *Birth Defects Research Part A: Clinical and Molecular Teratology* (2015) 103(6):527-35.

6. Tikkanen M. Placental abruption: epidemiology, risk factors and consequences. *Acta obstetricia et gynecologica Scandinavica* (2011) 90(2):140-9.

7. Blencowe H, Cousens S, Oestergaard MZ, Chou D, Moller A-B, Narwal R, et al. National, regional, and worldwide estimates of preterm birth rates in the year 2010 with time trends since 1990 for selected countries: a systematic analysis and implications. *The lancet* (2012) 379(9832):2162-72.

8. Cartlidge PH, Stewart JH. Effect of changing the stillbirth definition on evaluation of perinatal mortality rates. *The lancet* (1995) 346(8973):486-8.

9. Brown TM, Cueto M, Fee E. A transição de saúde pública'internacional'para'global'e a Organização Mundial da Saúde. *História, Ciências, Saúde-Manguinhos* (2006) 13(3):623-47.

10. Battaglia FC, Lubchenco LO. A practical classification of newborn infants by weight and gestational age. *The Journal of pediatrics* (1967) 71(2):159-63.

11. Casey BM, McIntire DD, Leveno KJ. The continuing value of the Apgar score for the assessment of newborn infants. *New England Journal of Medicine* (2001) 344(7):467-71.

12. Liu H, Liao J, Jiang Y, Zhang B, Yu H, Kang J, et al. Maternal exposure to fine particulate matter and the risk of fetal distress. (2019) 170:253-8.

13. Landmann E, Reiss I, Misselwitz B, Gortner L. Ponderal index for discrimination between symmetric and asymmetric growth restriction: percentiles for neonates from 30 weeks to 43 weeks of gestation. *The Journal of Maternal-Fetal & Neonatal Medicine* (2006) 19(3):157-60.

14. Yu M, Ping Z, Zhang S, He Y, Dong R, Guo X. The survey of birth defects rate based on birth registration system. *Chinese medical journal* (2015) 128(1):7.
